# Sirenomelia: A Rare Presentation

**Published:** 2012-01-01

**Authors:** K Ramesh Reddy, S Srinivas, Shiva Kumar, Surweshwar Reddy, Hari Prasad, G M Irfan

**Affiliations:** Niloufer Hospital, Institute of Child Health, Osmania Medical College, Hyderabad, A.P, India

**Keywords:** Sirenomelia, Mermaid syndrome, Caudal regression syndrome

## Abstract

We are presenting two cases of Sirenomelia (Mermaid Syndrome), which is an extreme example of the caudal regression syndrome. It invariably presents with lower limb fusion, sacral and pelvic bony anomalies, absent external genitalia, imperforate anus, and renal agenesis or dysgenesis. There are approximately 300 cases reported in the literature, 15% of which are associated with twinning, most often monozygotic. The syndrome of caudal regression is thought to be the result of injury to the caudal mesoderm early in gestation. One of our cases survived for 12 days after birth. This new born had an unusually high anorectal anomaly in which the colon was ending at the level of mid transverse colon, fused lower limbs and genital anomalies. Ultrasound of the abdomen revealed horseshoe kidney. Colostomy was performed on day 2 of life. The second case encountered was a stillborn baby on whom an autopsy was performed.

## INTRODUCTION

Sirenomelia is rare and fatal congenital anomaly with an incidence of 0.8 to 1 case per 1,00,000 births. Male to Female ratio being 3:1, the malformation sequence consists of varying degrees of lower limb fusion bearing a resemblance to the mermaid of ancient Greek mythology. Severe malformations of the gastrointestinal, genitourinary, cardiovascular and musculoskeletal systems are usually present. Oligohydromnios secondary to severe renal dysplasia is universal. The first medical description of Sirenomelia was by Rocheus and Polfyr way back in the sixteenth century. Duhamel in 1961 defined all the anomalies of mermaid syndrome and described it as the most severe form of caudal regression syndrome [1-3].

## CASE REPORT

**Case 1**

A newborn was referred to our institute on day one of life with fused lower limbs and absence of anal orifice and genitalia. The baby was born to non-consanguineous parents at 8 months of gestation. There was no history of exposure to any known teratogenic agent during the antenatal period. An antenatal scan revealed moderate to severe oligohydromnios and the fetal kidneys were not well visualized. There was no history of maternal diabetes. On examination, the neonate had a flat facial profile (Potter’s facies). The lower limbs were fused from the hip downwards and two feet were seen, (sirenomelia dipus); external genitalia were absent (Fig. 1). A small phallus like structure was seen in the back with an orifice from which urine was being passed, the anal orifice was absent. 2D Echocardiography revealed a large ventricular septal defect. Ultrasound abdomen and pelvis revealed fused pelvic kidney with altered echogenicity (suggestive of dysplasia), absent urinary bladder and ureters opening into the orifice at the back. Operative findings included colonic atresia distal to mid transverse colon with no distal colon, presence of uterus and ovaries and absence of urinary bladder (Fig. 2). An end colostomy was performed and child survived post operatively for 12days. Death was attributed to associated anomalies, i.e. renal and cardiac.

**Figure:**
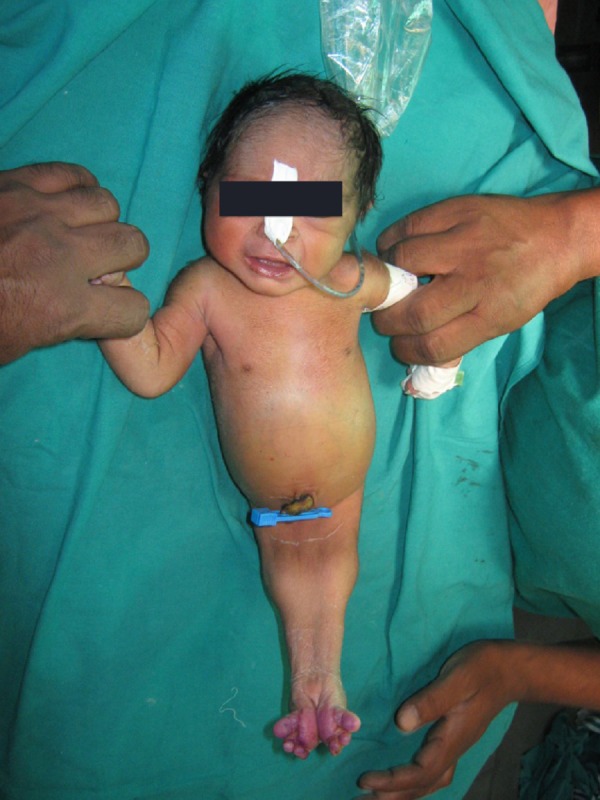
Figure 1: Mermaid

**Figure:**
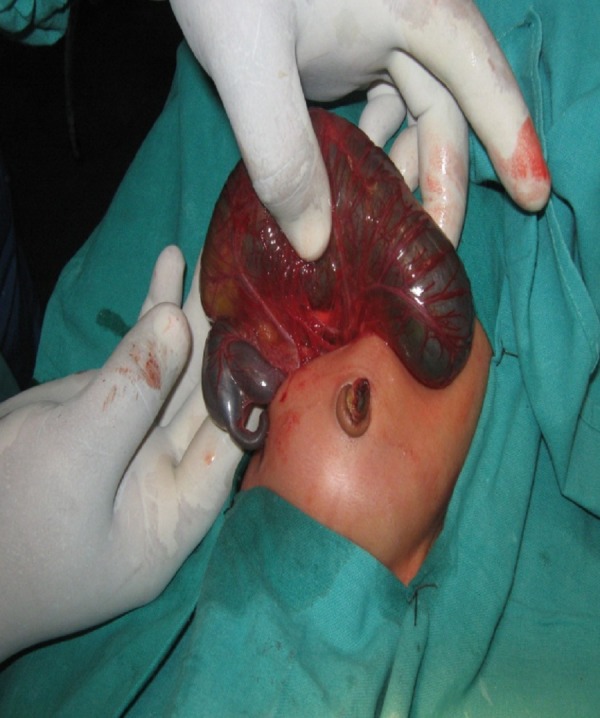
Figure 2: Mid Transverse Colon Atresia

**Case 2**

A 22-year-old primigravida delivered a stillborn baby at 8 months of gestation. The child was a product of non-consanguineous marriage. An antenatal scan showed severe oligohydromnios and the kidneys could not be visualized. The stillborn baby had Potter’s facies and sirenomelia dipus (lower limbs fused below hips; two feet were present) (Fig. 3). Autopsy revealed hypoplastic lungs, colonic atresia, absent right kidney and urinary bladder with small dysplastic left kidney. The external genitalia and anal orifice were absent.

**Figure:**
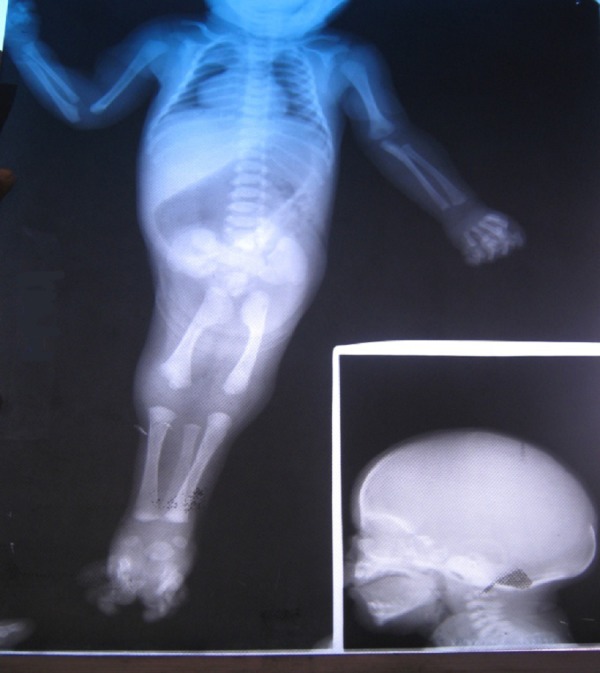
Figure 3: Babygram

## DISCUSSION

Sirenomelia is a rare congenital anomaly, till date approximately 300 cases have been reported in the literature. Most of these newborns were still born or died immediately after birth; death is usually due to renal agenesis, which is incompatible with life. Swader reported the first surviving infant in 1989. Till 2006, 6 cases of surviving infants with mermaid syndrome were reported. The etiology and pathogenesis of this malformation is unknown. Duhamel in 1961 stated that Sirenomelia and anorectal malformations represent the two extremes of a single comprehensive syndrome arising from an embryonal defect in the formation of the caudal region. He called it the Syndrome of Caudal Regression. Stevenson et al, dissected the abdominal vasculature of 11 cases of sirenomelia and demonstrated a pattern of vascular abnormality that explains the defect usually found in this condition. They demonstrated a single large artery (steal vessel) arising from the high intraabdominal cavity which diverts nutrients from the caudal end of embryo. There is a strong association between this syndrome and maternal diabetes; up to 22 % of fetuses with this anomaly are known to have diabetic mothers. However, neither of our cases had diabetic mothers. Fifteen percent of patients with sirenomelia have associated twinning, which is most often monozygotic [3,5-8].

Sirenomelia has been classified into three types according to the number of lower limb bones present:

1. Sirenomelia apus: No feet only one tibia and one femur.
2. Sirenomelia unipus : One foot, two femurs two tibia, and two fibula.
3. Sirenomelia dipus: Two feet and two fused legs giving the appearance of a flipper.

Sirenomelia dipus, also called as Mermaid syndrome, has the most favorable outcome. Survival of children with sirenomelia depends on the associated visceral anomalies, especially renal function, rather than the sirenomelia itself. Initial treatment of these newborns includes supportive care and diverting colostomy, later management of these infants includes a multidisciplinary surgical approach involving various specialties [5,9].

In conclusion, sirenomelia is a rare fatal congenital malformation with severe visceral anomalies that decide the survival. Fusion of the lower limbs, which is very obvious, is less fatal. Few surviving patients need a multidisciplinary approach of treatment.

## Footnotes

**Source of Support:** Nil

**Conflict of Interest:** None declared

